# Implementation of a Hybrid Teleneuropsychology Method to Assess Middle Aged and Older Adults During the COVID-19 Pandemic

**DOI:** 10.1093/arclin/acac037

**Published:** 2022-06-06

**Authors:** Amelia Ceslis, Lisa Mackenzie, Gail A Robinson

**Affiliations:** Neuropsychology Research Unit, School of Psychology, The University of Queensland, Brisbane, Australia; Queensland Brain Institute, The University of Queensland, Brisbane, Australia; Neuropsychology Research Unit, School of Psychology, The University of Queensland, Brisbane, Australia; Queensland Brain Institute, The University of Queensland, Brisbane, Australia

**Keywords:** Telehealth, Cognition, Neuropsychology assessment, Validity, In-clinic, Experience survey

## Abstract

**Objective:**

Hybrid teleneuropsychology has emerged as a useful assessment method to manage physical distancing requirements during the COVID-19 pandemic. We describe the development of a hybrid teleneuropsychology clinic and compare results of six neuropsychological tasks across testing modalities, as well as a participant experience survey.

**Method:**

Healthy middle-aged and older adults completed a face-to-face assessment two years previously. Participants either completed reassessment face-to-face or via the hybrid setup. Results were compared across time points and delivery modality.

**Results:**

There were no significant differences in scores at reassessment between face-to-face and a hybrid setup on nonverbal fluid intelligence, verbal memory, visual memory, language, working memory or verbal initiation. Retest reliability was moderate to excellent for verbal and visual memory, attention and naming. Results of an anonymous survey indicated that participants felt comfortable and established good rapport with the examiner.

**Conclusions:**

This hybrid method of teleneuropsychology can be used to obtain high quality and reliable results including on tasks yet to be evaluated for teleneuropsychology, including the Graded Naming Test and the Topographical Recognition Memory Test.

##  

Key factors of the COVID-19 pandemic like physical distancing, time restrictions and mask requirements have been difficult to manage with a traditional face-to-face (FTF) neuropsychology assessment set-up. Thus, many clinics and services have adopted teleneuropsychology (teleNP) methods for the first time (Bilder et al., 2020). According to Postal et al. (2021), there are three main models of teleNP: *in-clinic*, *remote* or *home*, and *hybrid*.

In-clinic teleNP utilizes videoconferencing to assess an individual situated in a remote clinic, usually with an assistant on site to aid the assessment (Appleman et al., 2021; Postal et al., 2021). This method has typically been used for those living in rural or regional communities or those whom have to travel long distances to attend an assessment. Although evidence suggests in-clinic teleNP is an appropriate method for assessment for older adults and those with cognitive impairment (Appleman et al., 2021; Cullum, Hynan, Grosch, Parikh, & Weiner, 2014; Cullum, Weiner, Gehrmann, & Hynan, 2006; Hildebrand, Chow, Williams, Nelson, & Wass, 2004; Marra, Hamlet, Bauer, & Bowers, 2020; Postal et al., 2021), the requirement of an assistant means this method is not necessarily suitable during a pandemic.

Home teleNP, involves videoconferencing into the individual’s home remotely via either a clinic or the neuropsychologist’s own home (Fox-Fuller et al., 2021; Postal et al., 2021). In a large survey study in the United States, home teleNP was reported as the most commonly utilized teleNP method by neuropsychologists and other health professionals engaging in teleNP during the COVID-19 pandemic (Fox-Fuller, Rizer, Andersen, & Sunderaraman, 2022). Home teleNP meets the social distancing requirements that cannot always be met through in-clinic teleNP and there is emerging evidence that a home teleNP can be a valid assessment method for adults with a wide range of neurological conditions (epilepsy: Tailby et al., 2020; neurodegenerative diseases: Parks, Davis, Spresser, Stroescu, & Ecklund-Johnson, 2021). Mahon, Webb, Snell, and Theadom (2021) reported the majority of healthy adults whom participated in both a traditional FTF assessment and a home teleNP assessment reported high satisfaction with the home teleNP experience, with most participants indicating they would be happy to engage in the home teleNP method again. Further, some participants with epilepsy have been reported as feeling more comfortable in a home environment when engaging in neuropsychology assessment compared to a traditional in-person assessment (Tailby et al., 2020).

While there are advantages to a home teleNP assessment during the COVID-19 pandemic, a home assessment adds additional challenges for a valid and reliable assessment. Fox-Fuller, Rizer, Andersen, and Sunderaraman (2022) surveyed neuropsychologists and other health professionals engaging in teleNP assessments and found issues with the home internet of the participant was the most common concern reported (88%). For older adults, a lack of familiarity with the required technology, lack of access to technology or high-speed internet, and other uncontrolled factors such as who else is in the room may also impact the assessment (Bilder et al., 2020; Fox-Fuller, Rizer, Andersen, & Sunderaraman, 2022; Mahon, Webb, Snell, & Theadom, 2021; Postal et al., 2021).

Hybrid teleNP is a broad term that describes a combination of traditional FTF and teleNP practices (Postal et al., 2021). Often, hybrid teleNP utilizes videoconferencing to assess individuals in the same facility but whom are situated in a different room to the neuropsychologist, although some tasks may still be completed FTF. Advantages of a hybrid teleNP method include more control over issues faced with home teleNP, and only requiring one neuropsychologist rather than an additional assistant used for in-clinic setups (Postal et al., 2021). While limited, a number of studies have found a hybrid teleNP model to be suitable for older adults and those with cognitive impairment (see Marra, Hamlet, Bauer, & Bowers, 2020 for a review). Cullum, Weiner, Gehrmann, and Hynan (2006) found comparable results for their 14 mild cognitive impairment (MCI) and Alzheimer’s disease (ad) participants in FTF and hybrid teleNP methods. An FTF and a teleNP assessment were completed on the same day (alternate versions of tests used) with the participant and neuropsychologist communicating between two rooms. There were no significant differences between testing methods for all ten tests used (cognitive screening, memory, attention, executive functions, language). Nine of the ten tests showed little difference between testing conditions and little bias between conditions. However, category fluency showed lower agreement between assessment methods (Cullum, Weiner, Gehrmann, & Hynan, 2006). In addition, Cullum, Hynan, Grosch, Parikh, and Weiner (2014) assessed MCI, ad (*n* = 83) and healthy control participants (*n* = 119) via FTF and teleNP. They found significant intraclass correlations between assessment methods for tests of memory, executive function, language and cognitive screening (Cullum, Hynan, Grosch, Parikh, & Weiner, 2014).

As Australia was initially less affected by the COVID-19 pandemic, a hybrid teleNP was reported as a good option for those whom still wanted to attend their assessment on site. We discuss how we successfully implemented a hybrid teleNP method at the University of Queensland Neuropsychology Research Clinic (UQNRC) by moving all neuropsychological testing to a hybrid teleNP model. We compared the results from standard FTF testing to our hybrid teleNP method on a group of middle and older aged adults whom had completed a neuropsychology assessment two years previously. We hypothesized there would not be a significant difference between the two testing methods. Results from a participant experience survey and limitations of this new method are also discussed.

## Method

### Clinic Adaptations and Testing Procedure

All participants that attended the UQNRC from June 2020 to December 2021 completed a hybrid teleNP research assessment. All set-up was completed before the participant arrived. Two adjacent testing rooms were used for each assessment. The rooms were set-up with the desktop computer (Dell, 60-cm screen) facing natural lighting with overhead lights switched off in the neuropsychologist’s room.

Communication was through the Zoom application with both computers using a hard-wired ethernet connection. Most neuropsychology tasks were administered via Zoom using either the share screen function or a document camera (IPEVO V4K Ultra High Definition; USB connection). As monitors were the same size, tasks projected from the neuropsychologist’s screen were reflected identically on the participant’s computer screen. The inbuilt monitor webcams were used for the video connection, with “High Definition” and “Speaker View” selected. With only two participants using Zoom, “Speaker View” means the other’s face is always large on their own screen (their own face is small at the top). The view stays stable throughout, no matter who is speaking. During tasks that use the “Share Screen” function, both the participant and examiner’s faces are shown small in the top right-hand corner of the screen (side-by-side mode enabled). External microphones (Logitech USB Desktop Microphone) were used in both rooms. Participants were instructed to participate in turn-taking when speaking over the audio-visual equipment to optimize communication and reduce overlay interference.

Acrylic drawers were placed on the participant’s desk, which contained questionnaires and other task stimuli. An audio recorder (Olympus VN-733PC) and video camera (Vado HD Pocket Cam) were placed in the participant room for tasks requiring transcription or post assessment analysis (e.g., spontaneous speech tasks, Hayling Sentence Completion Test, Praxis). Participants signed written consent to obtain these recordings. Any additional audio or visual material collected that was not relevant to the study was deleted. A lockable mobility chair was available for participants with mobility issues to ensure safety when the neuropsychologist was in a different room. Participants with mobility issues were instructed to stay in their seat until the neuropsychologist entered the room.

Tasks that were unable to be completed in a separate room (visual attention, visuomotor speed tasks) were completed at a desk, with the participant and neuropsychologist seated 1.5 meters opposite, a clear screen between and masks worn when required. Tasks completed face-to-face were not included in the present analysis.

### Participants

Healthy participants were recruited for the “Prospective Imaging Study of Ageing: Genes, Brain and Behavior” (PISA), a prospective cohort study investigating the biomarkers of early neuropathology and modifiable risk factors of ad (Lupton et al., 2021). Participants were predominately Caucasian with English as their first language. The PISA study involves a baseline neuropsychological assessment and a two-year reassessment. Neuropsychology scores for all study participants were reviewed against criteria for mild cognitive impairment (MCI; Albert et al., 2011) and Alzheimer’s disease (ad; McKhann et al., 2011) at baseline and reassessment by the study registered clinical neuropsychologists (full method can be found in Lupton et al., 2021).

All healthy PISA participants completed their baseline assessment FTF. Before COVID-19 lockdowns, 19 PISA participants completed their reassessment FTF. All other PISA participants having since completed their reassessment using our hybrid teleNP set-up (*N* = 141). To ensure the groups were not of extreme disproportionate size for our comparison analysis, we used a subclassification system to match Group 2 participants to Group 1 (Stuart, 2010). Group 2 participants were matched to Group 1 participants on age, sex, and education. Participants deemed healthy at baseline then met criteria for MCI or ad at reassessment were excluded from the analysis (*N* = 2, one participant from the FTF group and one participant from the hybrid teleNP group). Thus, there are two distinct groups of healthy participants: the first group (Group 1, *N* = 19) that completed both assessments FTF, and a second group (Group 2, *N* = 33) seen FTF at baseline and then via hybrid teleNP at reassessment.

There have also been 65 clinical participants that have attended for a hybrid teleNP assessment and diagnoses include Alzheimer’s disease, mild cognitive impairment, progressive supranuclear palsy, corticobasal syndrome, language dementias, frontotemporal dementia, and metabolic disorders. Clinical participants were referred by specialist consultants (e.g., neurologists, neuropsychiatrists) for a research assessment and neuropsychological opinion. However, clinical participants were not included in the hybrid teleNP analysis. The reason for this was twofold: firstly, many were seen only for a baseline assessment, and secondly, those whom completed a two-year follow-up likely had declined over the two-year period since their baseline assessment.

Approval for the study was granted by The University of Queensland Human Research Ethics Committee and all participants provided informed written consent.

### Neuropsychology Tasks and Assessment Comparison

We compared performance of the two healthy participant groups on eleven measures obtained from six neuropsychological tasks of nonverbal fluid intelligence (Wechsler Abbreviated Scale of Intelligence—Matrix Reasoning subtest; Wechsler, 2011), verbal memory (Rey Auditory Verbal Memory Test [AVLT]; total learning score, immediate free recall, delayed free recall, recognition hits; Ivnik et al., 1990), visual memory (Topographical Recognition Memory Test; Warrington, 1996), language (Graded Naming Test [GNT]; Warrington, 1997), auditory–verbal attention (Digit Span Forwards [DSF], Digit Span Backwards [DSB]; Wechsler, 2003), and executive functioning (Hayling Sentence Completion Test [HSCT]; Part 1 Initiation total reaction time, Global Error Score; Burgess & Shallice, 1997). Raw scores were used for all analyses, except for the HSCT Global Error Score that was based on Burgess and Shallice (1996; A type errors awarded 3 points, B type errors awarded 1 point; range = 0 to 45). Tasks completed FTF during the hybrid teleNP assessment (tasks of visuomotor processing speed, visual attention) were not included in our analysis.

All analyzed tasks were administered as per standardized instructions with the following adjustments made to fit our hybrid teleNP method. The Matrix Reasoning subtest, Graded Naming Test and Topographical Recognition Memory Test were presented to the participant using the document camera. As participants were unable to point to their answer on the Matrix Reasoning subtest, they were asked to say the number of their response. During the Topographical Recognition Memory Test, participants were instructed to look at the screen for the entire duration of the subtest, with the examiner able to track this to a degree by watching the participant on the side window (side-by-side mode selected). The Hayling Sentence Completion Test reaction times were always calculated post-assessment via the audio recorder to ensure these were recorded from when the participant heard the last word to guarantee accurate test interpretation (i.e., inaccurate timing affects the Scaled Scores of the task) and produce comparable performances. Test order was slightly different between time points and participants were not necessarily assessed by the same neuropsychologist at reassessment.

Log transformations were performed on RAVLT recognition hits (reverse log transformation) and HSCT Part 1 reaction times as data was skewed to ceiling and floor, respectively. Performance was compared for the two groups (Group 1: baseline and follow-up—FTF; Group 2: baseline—FTF, follow-up—hybrid teleNP) across Time (Time 1 [baseline] versus Time 2 [two-year follow-up]) and Group (FTF versus hybrid teleNP) using 2 × 2 mixed model ANOVAs. Follow-up *t*-tests were conducted to compare group performances at Time 2. One-way random-effects average-measurement intraclass correlations (ICCs) were used to analyze the test–retest agreement for Group 2 between Time 1 (FTF) and Time 2 (hybrid teleNP). ICCs were not conducted on Group 1 due to the smaller N, with the exception of the Matrix Reasoning (MR) subtest where we conducted a post-hoc ICC due to reviewer request.

### Participant Experience Survey

We were interested in participants’ experience of the hybrid teleNP assessment. A 7-item Participant Experience Survey using a 5-point Likert Scale was created for anonymous feedback. The items included: “I felt comfortable during my appointment,” “The procedures were clearly explained to me via phone/email,” “The quality of the sound / vision was,” I had good rapport with the clinician even though they were in a different room to me,” “My overall experience was positive.” The survey was provided at the conclusion of the hybrid teleNP assessment whereafter the participant placed the questionnaire in an unmarked envelope. To maintain anonymity, surveys were opened in clusters.

Sixty-three participants assessed at the UQNRC completed the Participant Experience Survey. Participants included a mix of the PISA healthy participants and clinical participants. Surveys were completely deidentified and therefore no demographic information was collected.

## Results

Groups were matched for age and education. Both groups comprised more female than male participants, reflective of the overall PISA study demographics (73% female; Lupton et al., 2021). There was no significant difference in the number of days between testing points between the two groups. See Table 1 for participant demographics.

**Table 1:** Demographic information

	Group 1 (FTF only) *M* (*SD*)	Group 2 (FTF/hybrid teleNP) *M* (*SD*)
*N*	19	33
Age (years) Range	62.5 (4.5) 53–69 years	62.8 (5.9) 50–73 years
Sex (M:F)	2:17	8:25
Education (years) Range	13.6 (3.0) 10–20 years	14.4 (2.8) 10–19 years
Days between assessments	843.1 (45.8)	860.6 (107.4)

Neuropsychology scores of the two groups are summarised in Table 2. Results indicated there were no significant interactions between Time and Group on any measure including Matrix Reasoning, *F*(1, 44) = 0.47, *p* = 0.495, partial eta squared = 0.011, AVLT verbal learning score, *F*(1,50) = 0.001, *p* = 0.983, partial eta squared <0.001, AVLT immediate free recall, *F*(1,50) = 0.15, *p* = 0.669, partial eta squared = 0.003, delayed free recall, *F*(1,50) = 1.18, *p* = 0.283, partial eta squared = 0.023, AVLT recognition hits, *F*(1,50) = 0.09, *p* = 0.769, partial eta squared = 0.002, topographical memory, *F*(1,50) = 0.001, *p* = 0.990, partial eta squared <0.001, DSF, *F*(1,50) = 0.22, *p* = 0.643, partial eta squared = 0.011, DSB, *F*(1,50) = 0.12, *p* = 0.731, partial eta squared = 0.002, GNT, *F*(1,50) = 1.36, *p* = 0.249, partial eta squared = 0.026, HSCT Part 1 reaction time, *F*(1,49) = 0.002, *p* = 0.969, partial eta squared <0.001, and HSCT global error score, *F*(1,49) = 0.03, *p* = 0.868, partial eta squared = 0.001.

A significant main effect of Time was found for AVLT verbal learning scores whereby there was an increase in learning scores at Time 2 compared to Time 1, *F*(1,50) = 9.20, *p* = 0.004, partial eta squared = 0.155, and a significant main effect of time was found for AVLT immediate free delayed recall score, whereby there was an increase in scores at Time 2 compared to Time 1, *F(*1,50) = 5.69, *p* = 0.021, partial eta squared = 0.102. There was also a significant main effect of Time found for HSCT global error score whereby there were lower error scores at Time 2 compared to Time 1, *F*(1,49) = 4.14, *p* = 0.047, partial eta squared = 0.078. However, there was no main effect of Time for any other measure including Matrix Reasoning, *F*(1,44) = 1.96, *p* = 0.169, partial eta squared = 0.043, AVLT delayed free recall, *F*(1,50) = 2.56, *p* = 0.116, partial eta squared = 0.049, AVLT recognition hits, *F*(1,50) = 0.11, *p* = 0.742, partial eta squared = 0.002, topographical memory, *F*(1,50) = 1.48, *p* = 0.230, partial eta squared = 0.066, DSF, *F*(1,50) = 3.06, *p* = 0.087, partial eta squared = 0.058, DSB, *F*(1,50) = 0.02, *p* = 0.892, partial eta squared <0.001, GNT, *F*(1,50) = 1.36, *p* = 0.249, partial eta squared = 0.026, and HSCT Part 1 reaction time, *F*(1,49) = 0.25, *p* = 0.620, partial eta squared = 0.005.

There was no significant main effect found for Group for any measures including Matrix Reasoning, *F*(1,44) = 2.04, *p* = 0.160, partial eta squared = 0.044, AVLT verbal learning score, *F*(1,50) = 0.76, *p* = 0.389, partial eta squared = 0.015, AVLT immediate free recall, *F*(1,50) = 2.48, *p* = 0.122, partial eta squared = 0.47, AVLT delayed free recall, *F*(1,50) = 1.80, *p* = 0.185, partial eta squared = 0.035, AVLT recognition hits, *F*(1,50) = 2.06, *p* = 0.158, partial eta squared = 0.040, topographical memory, *F*(1,50) = 0.03, *p* = 0.869, partial eta squared = 0.001, DSF, *F*(1,50) = 0.03, *p* = 0.874, partial eta squared = 0.001, DSB, *F*(1,50) = 0.47, *p* = 0.496, partial eta squared = 0.009, GNT, *F*(1,50) = 0.01, *p* = 0.941, partial eta squared = 0.000, HSCT Part1 reaction times, *F*(1,49) = 0.04, *p* = 0.843, partial eta squared = 0.001, and HSCT Global Error Score, *F*(1,49) = 0.03, *p* = 0.875, partial eta squared = 0.001.

**Table 2:** Group comparison on neuropsychology tasks across testing format

		Group 1		Group 2	
		Time 1	Time 2	Time 1	Time 2
Neuropsychology measures	*M* (norm)	FTF *M* (*SD*)	FTF *M* (*SD*)	FTF *M* (*SD*)	Hybrid teleNP *M* (*SD*)
WASI-II MR/30)	17.5	18.5 (3.3)	18.1 (3.5)	20.0 (2.7)	18.9 (3.1)
AVLT learning score (/75)	49.7	48.3 (8.5)	51.4 (8.9)^**a^	50.3 (9.0)	53.4 (8.4)^**a^
AVLT immediate recall (/15)	10.0	9.1 (2.5)	10.2 (3.3)^*a^	10.4(2.6)	11.2 (2.9)^*a^
AVLT delayed recall (/15)	9.9	9.1 (2.5)	10.1 (3.5)	10.6 (2.9)	10.7 (3.1)
AVLT recognition hits (/15)	13.9	13.4 (1.6)	13.6 (1.2)	14.0 (1.4)	13.9 (1.3)
Topographical memory (/30)	23	26.1 (2.6)	25.6 (2.4)	26.2 (3.0)	25.7 (2.8)
Digit span forwards (/16)	10	11.2 (2.7)	10.9 (2.5)	11.2 (2.7)	10.7 (2.6)
Digit san backwards (/16)	8	9.2 (2.6)	9.2 (2.8)	9.7 (2.5)	9.6 (2.0)
GNT (/30)	20	22.3 (3.5)	22.3 (2.5)	22.1 (3.1)	22.7 (3.2)
HSCT Part 1 RT (seconds)	4	4.6 (3.8)	4.5 (4.7)	5.9 (7.1)	4.1 (3.1)
HSCT global error (/45)	NA	5.2 (4.7)	3.68 (3.9)^*a^	5.2 (2.9)	3.9 (4.2)^*a^

FTF = face-to-face; *M* (norm) = mean or score at 50th percentile obtained from normative sample. MR = Matrix Reasoning; AVLT = Auditory Verbal Learning Test; GNT = Graded Naming Test; HSCT = Hayling Sentence Completion Test. RT = reaction time; NA = not applicable. ^a^ Main effect of Time. ^*^p < 0.05. ^*^^*^p < 0.01.

Time 2 follow-up *t*-tests revealed there were no significant differences in scores between groups for any measure including Matrix Reasoning, *t*(44) = −0.77, *p* = 0.447, AVLT verbal learning score, *t*(50) = −0.80, *p* = 0.430, AVLT immediate free recall, *t*(50) = −1.12, *p* = 0.266, AVLT delayed free recall, *t*(50) = −0.72, *p* = 0.473, AVLT recognition hits, *t*(50) = 1.217, *p* = 0.229, topographical memory, *t*(50) = −0.15, *p* = 0.879, DSF, *t*(50) = 0.31, *p* = 0.757, DSB, *t*(50) = −0.54, *p* = 0.590, GNT, *t*(50) = −0.45, *p* = 0.656, HSCT Part 1 reaction times, *t*(49) = 0.13, *p* = 0.897, and HSCT Global Error Score, *t*(49) = −0.22, *p* = 0.831.

For Group 2, test–retest statistics indicated there was moderate level of consistency across time for topographical memory, ICC(1) = 0.53, *p* = 0.012, and AVLT recognition hits, ICC(1) = 0.60, *p = 0*.001, a good level of consistency across time for AVLT verbal learning scores, ICC(1) = 0.76, *p* < 0.001, AVLT immediate free recall, ICC(1) = 0.74, *p* < 0.001, AVLT delayed free recall, ICC(1) = 0.87, *p* < 0.001,DSF, ICC(1) = 0.87, *p* < 0.001, DSB, ICC(1) = 0.82, *p <* 0.001, and an excellent level of consistency across time for GNT scores, ICC(1) = 0.91, *p* < 0.001. However, there was a low level of consistency for MR scores, ICC(1) = 0.24, *p* = 0.226, HSCT Part 1 reaction times, ICC(1) = 0.06, *p = 0*.377, and HSCT Global Error Score, ICC (1) = 0.32, *p* = 0.028. For Group 1, post-hoc test–retest statistics indicated a good level of consistency across time for MR scores, ICC(1) = 0.84, *p* = 0.000.

### Participant Experience Survey

Results indicated that all participants felt comfortable during their appointment. Procedures were clearly explained either via phone or email to all but one participant. The sound was either good or excellent quality for 98% of survey participants. The quality of the video was either good or excellent quality for 94% of survey participants. All survey participants reported experiencing good or excellent rapport and 92% of survey participants reported an overall good or excellent experience. While five participants strongly disagreed that their experience was positive, these responses were all incongruent with previous item responses that were all positively selected. See Table 3 for survey results.

**Table 3:** Participant Experience Survey (*N* = 63)

	Strongly agree	Agree	Neutral	Disagree	Strongly disagree
	*N* (%)	*N* (%)	*N* (%)	*N* (%)	*N* (%)
Felt comfortable	56 (89%)	7 (11%)	0	0	0
Procedures clearly explained before	53 (84%)	9 (14%)	1	0	0
Less worried about infection control	11 (17%)	15 (24%)	32 (51%)	0	5 (8%)
Overall experience was positive	50 (79%)	8 (13%)	0	0	5 (8%)^^^
	Excellent	Good	Adequate	Adequate at times	Very poor
The sound quality was	50 (79%)	12 (19%)	0	0	1^#^
The video quality was	51 (81%)	8 (13%)	1	0	1
		Most of the time	Neutral	Some of the time	Not at all
Good rapport with the clinician^a^	60 (95%)	2	0	0	0

^Incongruent response to other items. ^#^Participant indicated hearing issues. ^a^One participant indicated both “all of the time” and “most of the time” and their split response was not included.

## Discussion

We successfully established a hybrid model of teleNP that substantially reduced contact from standard FTF assessment methods. Results of the follow-up t-tests indicated there were no significant score differences between the FTF or hybrid teleNP groups at Time 2 on any of the eleven key neuropsychology measures selected from attention, memory, language, intelligence and executive function domains in our healthy adult sample.

There were no significant group differences in scores between baseline and two-year follow-up assessments on eight of the eleven neuropsychology measures. While verbal learning and immediate free recall scores were significantly higher at Time 2, scores were not significantly different between the FTF and hybrid teleNP groups. In addition, participants had highly consistent verbal learning scores across time despite some being reassessed using a different method. As participants were retested on the same version of the Auditory Verbal Learning Test, this overall increase in scores is likely attributable to practice effects (Strauss, Sherman, & Spreen, 2006).

In regard to Group 2 (reassessed via hybrid teleNP), moderate to excellent retest consistencies were observed for eight of the eleven measures selected including verbal memory, visual memory, auditory–verbal attention, and naming. The good level of agreement found for the Auditory Verbal Learning Test and Digit Span is in line with previous research assessing healthy adult and older adult participants using hybrid teleNP methods (Cullum, Hynan, Grosch, Parikh, & Weiner, 2014; Wadsworth et al., 2016; Hildebrand et al., 2014). Further, the retest timeframes from these hybrid teleNP studies have been established on the same day using alternate test versions (Cullum, Hynan, Grosch, Parikh, & Weiner, 2014; Wadsworth et al., 2016) or two–four weeks apart (Hildebrand, Chow, Williams, Nelson, & Wass, 2004), whereas we have established good reliability using hybrid teleNP methods over a two-year period.

In addition, there was an excellent level of agreement between Time 1 and Time 2 for the Graded Naming Test, despite participants in Group 2 being assessed over hybrid teleNP at Time 2. Previous research has found that the Graded Naming Test has very good retest reliability in person (Bird, Papadopoulou, Ricciardelli, Rossor, & Cipolotti, 2004); however, this is the first instance we know of that has reported the reliability of the Graded Naming Test using teleNP.

Visual memory has rarely been evaluated for teleNP methods. There was a moderate level of agreement for Group 2 between Time 1 and Time 2 for topographical memory and this is the first instance to our knowledge that has reported the reliability of the Topographical Recognition Memory Test using teleNP. Our results are in line with Chapman, Gardner, Ponsford, Cadilhac, and Stolwyk (2021) whom found a moderate level of agreement for WMS-IV Visual Reproduction and a good level of agreement for the Rey Complex Figure Test delayed recall between FTF and a remote teleNP assessment. However, both the Visual Reproduction and Rey Complex Figure Test require participants to draw their responses, which requires either trust that the participant can complete the task correctly without the examiner observing, an assistant in the room to observe, or a second camera that points to the participant’s drawing. The Topographical Recognition Memory Test may offer an advantage over these issues.

A meta-analysis of teleNP use in adult populations found no significant change in scores between FTF and teleNP setups, although there was a large heterogeneity reported between studies (Brearly et al., 2017). Indeed, while there were no main effects of time or group for Matrix Reasoning, intraclass correlations revealed a low level of agreement for Group 2 scores between Time 1 and Time 2. This is in contrast to a very high intraclass correlation (0.90) reported for Matrix Reasoning in a study of 30 healthy adults (18–40 years; Mahon, Webb, Snell, & Theadom, 2021). One possibility for this difference may be due to the test–retest timeframe, as there was an average of 7 days between assessments in the Mahon, Webb, Snell, and Theadom (2021) study whereas there was two-year reassessment period in ours. Further, follow-up *t*-tests revealed no differences between groups at Time 2, indicating no difference in scores between testing modality. This is in line with previous research using the first edition on the WASI that also found no group differences on the perceptual reasoning index (which includes the Matrix Reasoning subtest) when healthy adult participants were reassessed using hybrid teleNP methods (Temple, Drummond, Valiquette, & Jozsvai, 2010). However, there was a good level of agreement for Group 1 scores between Time 1 and Time 2, where the assessments were also completed two-years apart. There is a possibility that the teleNP administration for this subtest is less robust than the other measures used in our study and further investigation in this subtest may be required.

Few studies have evaluated executive functions beyond verbal fluency and clock drawing tasks (Marra, Hamlet, Bauer, & Bowers, 2020). This is the first instance we are aware of that the Hayling Sentence Completion Test has been evaluated for teleNP assessment. We assessed verbal initiation on the Hayling Sentence Completion Test Part 1 using the total reaction time and Global Error Score and found no main effects of group, or group differences at Time 2. While intraclass correlations were low, unlike scores attached to knowledge or reasoning, reaction times are likely going to vary between assessments, and the split-half reliability for Part 1 reaction times reported in the test manual for healthy participants is also low (0.35, *p < 0.*001; Burgess & Shallice, 1997). Further, executive function tasks that rely on novelty, like the Hayling Sentence Completion Test, perhaps are less reliable across assessments. Importantly, reaction times and error scores were comparable across testing methods at reassessment.

Two healthy participants converted to an MCI diagnosis at reassessment. Review of baseline scores indicated both participants performed poorly at baseline (<5th percentile on three cognitive tasks) and as such they were labelled as participants to watch for conversion at reassessment. While there may be an inherent factor in the hybrid teleNP set-up that enhances poorer performances in some, this is unlikely to be the reason these two met criteria at follow-up, particularly for the participant who was reassessed face-to-face.

Subjectively, participants with mild to moderate dementia coped well; however, participants with very severe dementia or severe visual deficits had more difficulty completing nonverbal tasks over video-link. Two participants with Alzheimer’s disease (Clinical Dementia Rating score of 3; Morris, 1997) were unable to complete any of the tasks in a separate room. However, using a hybrid teleNP method does allow for flexibility to test in the same room if needed.

Importantly, previous research using teleNP assessments has found patterns of expected impairments in those with mild or major neurocognitive disorder and epilepsy (Parks, Davis, Spresser, Stroescu, & Ecklund-Johnson, 2021; Tailby et al., 2020). Using a home teleNP set-up, Parks, Davis, Spresser, Stroescu, and Ecklund-Johnson (2021) found significant differences in performances between healthy controls and those with Alzheimer’s disease on tests of memory, naming and executive functions, which is an expected pattern of results. Likewise, participants with epilepsy that were assessed via teleNP performed poorer on cognitive domains that were expected to be impacted, including memory, working memory, language, executive functions, and processing speed (Tailby et al., 2020).

Results from our survey indicated that the experience was positive and well tolerated for the majority of participants surveyed, and good rapport was established in all cases. This is consistent with research from in-clinic teleNP studies. Hildebrand et al. (2014) survey results indicated in their group of healthy older adult participants 17% preferred teleNP and 39% had no preference to testing condition. An in-clinic teleNP method for assessment of regional veterans found participant satisfaction of a full teleNP neuropsychological assessment was high for those assessed in a remote clinic (Appleman et al., 2021).

Advantages of the hybrid teleNP assessment is individuals with cognitive difficulties are not required to trouble-shoot technical problems. It also allows for control of conditions as well as reliable and consistent equipment across all participants and sessions. Qualitative observations are not forgone as some face-to-face contact remains and therefore limits of clinical judgements as a consequence of a remote teleNP assessment do not apply to the same degree (Sozzi et al., 2020). Outside of the current COVID-19 pandemic, an advantage may be that vulnerable participants or vulnerable neuropsychologists are still able to complete a reliable neuropsychological assessment. Further, an established hybrid teleNP set-up is already in place should there be future virus outbreaks.

On the other hand, hybrid teleNP requires all participants to travel and attend the clinic, which may be a disadvantage to those whom do not live locally, and while exposure is limited there is still a small face-to-face component of the assessment. Space (i.e., two testing rooms for one assessment) may also be an issue for some clinics.

A limitation of our study is that we did not collect data on participant experience before COVID-19 and therefore we have no data on our participant’s experience of traditional FTF testing. We also noted five participants whom strongly disagreed that their experience was positive, which was incongruent with previous item responses that were all positively selected. The final survey question “my overall experience was positive” was reverse scored and perhaps this accounts for these inconsistencies. Alternatively, it may be that while the quality of the equipment was sound, some participants rated their overall experience as negative. Further, we have no data on the response rate of the survey, and perhaps it was only those whom had a positive experience responded.

In summary, we successfully incorporated a hybrid model of teleNP into the UQNRC and found no significant differences between FTF or hybrid teleNP groups on tasks of attention, memory, language, intelligence and executive function in a group of healthy middle and older aged adults. In regard to Group 2 (reassessed via hybrid teleNP), moderate to excellent retest consistencies were observed for eight of the eleven measures selected including verbal memory, visual memory, auditory–verbal attention, and naming. This is the first instance we are aware of that has evaluated the reliability of the Graded Naming Test, Topographical Recognition Memory Test and Hayling Sentence Completion Test for teleNP. All surveyed participants established rapport with the clinician in the different room and the experience was generally well tolerated. Given this was the first time some tasks had been evaluated for teleNP, future research should address the results of clinical participants completing a hybrid teleNP assessment.
